# *Neonauclea reticulata* (Havil.) Merr Stimulates Skin Regeneration after UVB Exposure via ROS Scavenging and Modulation of the MAPK/MMPs/Collagen Pathway

**DOI:** 10.1155/2013/324864

**Published:** 2013-06-15

**Authors:** Hsiu-Mei Chiang, Hsin-Chun Chen, Hua-Hsien Chiu, Chien-Wen Chen, Ssu-Meng Wang, Kuo-Ching Wen

**Affiliations:** ^1^Department of Cosmeceutics, China Medical University, Taichung 404, Taiwan; ^2^Center for Biomedical Technology Research and Development, Fooyin University, Kaohsiung 83102, Taiwan

## Abstract

In this study, we investigated whether the protective effects of *Neonauclea reticulata* water extract against ultraviolet B (UVB) irradiation in human skin fibroblast cell cultures (Hs68) are governed by its ability to protect against oxidative stress and expression of matrix metalloproteinases (MMPs). We found that *Neonauclea reticulata* extract exhibited DPPH scavenging activity and inhibited AAPH-induced haemolysis of erythrocytes in a dose- and time-dependent manner. We also found that pretreatment of fibroblasts with *Neonauclea reticulata* water extract resulted in markedly lower levels of MMP-1, -3, and -9 expressions. Furthermore, our results indicate that *Neonauclea reticulata* extract inhibits the expression of MMPs by inhibiting ERK, JNK, and p38 phosphorylation. Our results also demonstrate that treatment with *Neonauclea reticulata* extract protects against UVB-induced depletion of collagen. In addition, *Neonauclea reticulata* extract did not have a cytotoxic effect. These findings indicate that the antioxidant activity of *Neonauclea reticulata* extract resulted in inhibition of MMP-1, -3, and -9 expressions and in increased levels of collagen activity. Our results suggest that *Neonauclea reticulata* extract can protect against photoaging.

## 1. Introduction

Ultraviolet (UV) irradiation causes hazardous effects on the structure and function of skin, including sunburn, immune suppression, cancer, and photoaging [1, 2]. UV-induced oxidative damage and induction of matrix metalloproteinases (MMPs) have been implicated in skin photoaging. Photoaging is thought to occur by continuous damage to the collagenous extracellular matrix (ECM) that comprises the dermal connective tissue [3], and histological studies have demonstrated that the alterations are found in the dermal layer of photoaged skin. Collagen is the major insoluble fibrous protein in the extracellular matrix and in connective tissue, and type I collagen is the most abundant subtype of collagen. Collagen is synthesized primarily by fibroblasts residing within the dermis and is responsible for conferring strength and elasticity of skin [4]. Disorganization, fragmentation, and dispersion of collagen bundles are prominent features of photodamaged human skin. 

Ultraviolet B (UVB) irradiation stimulates the generation of reactive oxygen species (ROS) and induces the overexpression of MMP-1, -3, and -9 in human fibroblasts, resulting in the destruction of collagen and, hence, wrinkle formation and sagging skin [5–8]. Increased levels of ROS are known to alter the structure and function of genes and proteins in skin and MMPs, which are involved in extracellular matrix (ECM) remodelling, play important roles in cell migration, skin ulceration, and photoaging [9].

The results from recent studies have shown that phytochemicals such as polyphenols are excellent antioxidant and antiphotoaging agents [7, 10, 11]. Polyphenols and flavonoids are abundant in fruits, vegetables, green tea, and red wine and possess a variety of biological activities including antioxidants and the ability to inhibit the expression of MMPs in dermal fibroblasts. *Neonauclea reticulata* (Havil.) Merr is a member of the flavonoid-rich Rubiaceae family of flowering plants. In our previous studies, we found that *Coffea arabica* and *Ixora parviflora*, also members of the Rubiaceae family, were rich in polyphenols content and exhibited antiphotoaging activity by inhibiting the expression of MMPs and mitogen-activated protein kinases (MAPKs) [7, 8]. Therefore, we expected that plants who belonged to Rubiaceae family, such as *Neonauclea reticulate*, may exhibit similar activity. In our preliminary study, *Neonauclea reticulata* extract showed high total phenolic content and good ROS scavenging activity, suggesting that *Neonauclea reticulata* extract might be effective against UVB-induced photoaging. The aim of this study was to investigate the mechanisms through which *Neonauclea reticulata* extract protects against UVB-induced oxidative stress and photoaging in human skin fibroblast cell cultures. In addition, in our knowledge, this is the first report of the biological benefits of *Neonauclea reticulata*.

## 2. Materials and Methods

### 2.1. Materials

Human foreskin fibroblasts were obtained from the Bioresource Collection and Research Center (Hsinchu, Taiwan). Gelatin, agarose, hydrochloric acid, methanol, dimethyl sulfoxide (DMSO), doxycycline hyclate, calcium chloride (CaCl_2_), DL-dithiothreitol, and Folin-Ciocalteu reagent were purchased from Sigma-Aldrich Chemicals (St. Louis, MO, USA). Fetal bovine serum (FBS), penicillin-streptomycin, trypsin-EDTA, and Dulbecco's Modified Eagle's Medium (DMEM) were purchased from Gibco, Invitrogen (Carlsbad, CA, USA). Coomassie blue R-250, dibasic sodium phosphate, igepal CA-630, tris, and 3-(4,5-dimethylthiazol-2-yl)-2,5-diphenyltetrazolium bromide (MTT) were purchased from USB (Cleveland, OH, USA). Collagenase was purchased from Calbiochem, Merck (Darmstadt, Germany). Fluorogenic peptide substrate I was purchased from R&D Systems (Wiesbaden, Germany), and Bradford reagent was supplied by Bio-Rad Laboratories (Hercules, CA, USA). Donkey anti-goat IgG-HRP, ERK 1 (C-16), JNK1 (G-13), MMP-1 (L-20), MMP-3 (1B4), MMP-9 (6-6B), p38 (A-12), p-p38 (Thr 180/Tyr 182)-R, p-JNK (Thr 183/Tyr 185), and p-ERK 1/2 (Thr 202/Tyr 204) were purchased from Santa Cruz Biotechnology, Inc. (CA, USA). All other chemicals used were of high purity biochemistry grade.

### 2.2. Preparation of *Neonauclea reticulata* Extract

Fresh leaves of *Neonauclea reticulata* were harvested and identified at the National Museum of Natural Science, Taichung, Taiwan. The leaves were identified by macroscopic and microscopic examination by Dr. T. Y. Aleck Yang, and the voucher specimen was deposited in the National Museum of Natural Science (no. TNM BS 00599). The leaves were dried, ground, and then extracted twice with a 30-fold volume of water or methanol ultrasonically for 1 h. The supernatant was filtered, and the filtrate was evaporated to dryness in vacuo. The *Neonauclea reticulata* extract was stored at −20°C before use.

### 2.3. Total Phenolic Content of *Neonauclea reticulata* Extract

Total phenolic content was determined by the Folin-Ciocalteu reaction as reported previously [7]. Briefly, a mixture of *Neonauclea reticulata* extract and Folin-Ciocalteu phenol reagent was prepared, and then sodium carbonate was added to the mixture. The resulting blue complex was then measured at 760 nm. Gallic acid was used as a standard for the calibration curve. The contents of phenolic compounds were expressed as mg gallic acid equivalent/g dry weight. The dry weight indicated was *Neonauclea reticulata* leaves dry weight.

### 2.4. The Antioxidant Effects of *Neonauclea reticulata* Preparations

#### 2.4.1. DPPH Radical Scavenging Activity

In this assay, ascorbic acid (50 *μ*g/mL) was used as a positive control. Reaction mixtures containing DPPH and serial dilutions of sample (amounts of sample ranging from 25 to 1000 *μ*g/mL) were placed in a 96-well microplate at room temperature in the dark for 30 min. After incubation, the absorbance was read at 517 nm by ELISA. Scavenging activity was determined by the following:

(1) $$\begin{array}{c} \text{scavenging}\;\;\text{activity}\;\;\left(\%\right)=\left[1-\left(\frac{A_{\text{sample}}}{A_{\text{control}}}\right)\right]\times 100. \end{array}$$

#### 2.4.2. Preparation of Erythrocyte Suspensions and Hemolysis Assay

Blood was obtained from male SD rats via cardiopuncture, and whole blood was collected in EDTA-containing tubes. This animal study adhered to The Guidebook for the Care and Use of Laboratory Animals (published by The Chinese Society for Laboratory Animal Science, Taiwan). The erythrocytes were isolated by centrifugation, washed with PBS, and then resuspended to the desired hematocrit level. In order to induce free radical chain oxidation in the erythrocytes, aqueous peroxyl radicals were generated by thermal decomposition of AAPH in oxygen. A erythrocyte suspension at 5% hematocrit was incubated with PBS (control) or preincubated with *Neonauclea reticulata* extract (10–50 *μ*g/mL), followed by incubation with or without AAPH in PBS. This reaction mixture was shaken gently while being incubated for a fixed interval at 37°C. The reaction mixture was removed and centrifuged at 3000 ×g for 2 min, with absorbance of the supernatant determined at 540 nm. Reference values were determined using the same volume of erythrocytes in a hypotonic buffer. The hemolysis percentage was calculated using the formula [(*A*_sample_/*A*_control_)] × 100.

#### 2.4.3. Peroxide Scavenging Assay

The ability of *Neonauclea reticulata* extract to scavenge H_2_O_2_ was determined spectrophotometrically as previously described [12]. Briefly, a 20 mM solution of H_2_O_2_ was prepared in PBS (pH 7.4), added to various concentrations of *Neonauclea reticulata* extract that had been dissolved in methanol (50 to 2000 *μ*g/mL), and then allowed to stand at room temperature in the dark. The absorption was measured at 230 nm using an ELISA reader (Tecan, Grodig, Austria). The H_2_O_2_ scavenging activity of *Neonauclea reticulata* extract was determined by the following:

(2) scavenging  effect  (%)  =(Acontrol  at  230 nm−Asample  at  230 nmAcontrol  at  230 nm)×100.

### 2.5. MMP Activity Assay by Fluorescent Gelatin

The MMP activity in samples exposed to *Neonauclea reticulata* was assessed using a fluorescent method. Various concentrations of *Neonauclea reticulata* extract were tested for its ability to digest a synthetic fluorogenic substrate. Each concentration of *Neonauclea reticulata* extract was incubated with substrate at 37°C. Fluorescence intensity was measured at 320 nm (excitation) and 405 nm (emission) with a fluorescence reader. 

### 2.6. Cell Culture

Human foreskin fibroblasts (Hs68) cells were plated in a 10 cm dish and grown in DMEM supplemented with 10% FBS, 100 U/mL penicillin, and 100 U/mL streptomycin at 37°C in 5% CO_2_ humidified air. The cells were subcultured following trypsinization. For UVB irradiation, the medium was removed and washed in PBS. All UVB irradiations were performed under a thin layer of PBS. After UVB irradiation, cells were rinsed twice with PBS and incubated in fresh culture media without serum in the presence of *Neonauclea reticulata* extract for a further 24 h.

### 2.7. UVB Irradiation Dose

The source of UVB radiation was a UV lamp with a digital controller to regulate UV dosage (CL-1000 M, UVP, USA). The peak emission was recorded at 302 nm. The UVB irradiation dose was 40 mJ/cm^2^ (exposure time was 15 seconds). According to the results of our preliminary study, this UV dose induces the expression of MMPs and MAPKs but does not significantly affect cell viability (data not shown). After UVB irradiation, PBS was replaced with a serum-free medium, and cells were incubated for 24 h before subjecting them to the thiazolyl blue tetrazolium bromide (MTT) and MMP assays.

### 2.8. Cell Viability Test

Fibroblasts were plated at a density of 10^4^ cells/well in 96-well plates per 100 *μ*L medium and treated with 50 *μ*L of various concentrations of *Neonauclea reticulata* extract dissolved in DMEM and a small amount of DMSO for 24 h (the final concentration of DMSO was lower than 0.1%). The cytotoxic effect of *Neonauclea reticulata* extract on cells exposed to UVB and on cells that were unexposed to UVB irradiation was evaluated in cells that had been cultured for 3 h in MTT solution. Metabolic activity was quantified by measuring light absorbance at 570 nm (Tecan, Grodig, Austria).

### 2.9. Fluorescence Assay of Intracellular ROS

This assay is based on the use of an established nonfluorescent (DCFDA)/fluorescent (DCF) system that measures the levels of ROS, which are in turn responsible for the generation of fluorescence [12]. Hs68 cells were seeded in a 24-well plate at a density of 10^5^ cells/well for 24 h, rinsed once with 0.5 mL PBS, and then exposed to UVB irradiation (302 nm, CL-1000 M, UVP, USA). The UVB irradiation dose was 80 mJ/cm^2^ (exposure time was about 30 seconds). After that, PBS was removed, and then various concentrations of *Neonauclea reticulata *extract (1, 5, 10, and 50 *μ*g/mL) that had been prepared in serum-free DMEM were added. Cells were then allowed to incubate for 24 h at 37°C. The cells were then incubated for 30 min in the presence of 10 *μ*M DCFDA. After that, the DMEM was removed and the cells were washed twice with PBS. The cells were then covered with PBS. Images were observed under a fluorescence microscope (Leica DMIL, German), and the fluorescence (emission 520 nm, excitation 488 nm) was measured using a microplate reader (Thermo Electron Corporation, Vantaa, Finland). The following equation was used to calculate the relative fluorescence:

(3) $$\begin{array}{c} \text{relative}\;\;\text{fluorescence}\;\;\left(\%\right)=\left(\frac{A_{\text{control}}-A_{\text{sample}}}{A_{\text{control}}}\right)\times 100. \end{array}$$

### 2.10. Immunoblot Analysis

Cells were harvested and homogenized with lysis buffer [7]. Equal amounts of protein were separated on 10% SDS-PAGE gels and then transferred to a PVDF membrane (Hybond ECL, Amersham Pharmacia Biotech Inc., Piscataway, NJ, USA). Blots were blocked with nonfat milk in TBS buffer containing 0.05% Tween 20 (TBST). The membrane was incubated overnight at 4°C with specific antibodies. The antibodies comprised goat polyclonal antibodies against MMP-1 (1 : 500) and type I procollagen (1 : 500) and mouse polyclonal antibodies against MMP-3 (1 : 500), MMP-9 (1 : 500), ERK (1 : 500), JNK (1 : 500), p38 (1 : 500), p-ERK (1 : 500), p-JNK (1 : 500), and p-p38 (1 : 500) (Santa Cruz Biotechnology, Inc.). The membranes were washed with TBST, and the blots were then incubated with the corresponding conjugated anti-immunoglobulin G-horseradish peroxidase (Santa Cruz Biotechnology Inc.). Immunoreactive proteins were detected with the ECL Western blotting detection system (Fujifilm, LAS-4000). Signal strengths were quantified using a densitometric program (multi Gauge V2.2). 

### 2.11. Measurement of Total Collagen Synthesis

Total collagen synthesis in fibroblasts after UVB exposure was measured by the Sircol soluble collagen assay kit (Biocolor Ltd., UK) according to the manufacturer's protocol. Briefly, cell culture medium was mixed with Sircol dye reagent and incubated at room temperature for 30 min. After centrifugation, ice-cold acid-salt washing reagent was added to the precipitate, and then the mixture was centrifuged. The precipitate was dissolved with Alka reagent, and the absorption was determined at 555 nm by an ELISA reader (Tecan, Grodig, Austria).

### 2.12. Statistical Analysis

Results are expressed as mean ± SD. Differences between groups were analyzed by ANOVA followed by the Scheffe's test. A *p* value <0.05 was considered to represent statistical significance. 

## 3. Results

### 3.1. The Extraction Yield and Total Phenolic Content of *Neonauclea reticulata* Preparations

The extraction yield of *Neonauclea reticulata* leaves was 5.6% by methanol and 17.0% by water. The total phenolic content in the extract was determined by the Folin-Ciocalteu method. The total phenolic content, expressed as *μ*g gallic acid equivalent per mg of dry weight (*Neonauclea reticulata* leaves) was 24.2 ± 1.6 *μ*g/mg in the water extract and 9.2 ± 0.6 *μ*g/mg in the methanol extract.

### 3.2. Fluorometric Analysis of the Inhibitory Effect of *Neonauclea reticulata* Preparations on Bacterial Collagenase-1

**Figure 1:**
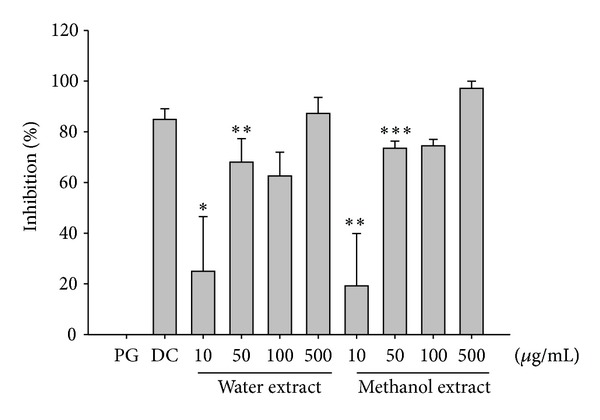
Collagenase fluorescence assay of methanol and water extracts of *Neonauclea reticulata extract *(1000 *μ*g/mL). Methanol and water extracts of *Neonauclea reticulata* significantly reduced collagenase activity. (*P < 0.05; **P < 0.01; ***P < 0.001).

In order to elucidate the inhibitory effect of *Neonauclea reticulata* extract on bacterial collagenase-1, fluorescence-conjugated gelatin was used, and the results were compared with the positive control, namely, doxycycline hyclate (DC). In this study, a fluorescence-conjugated substrate was incubated with bacterial collagenase-1 for 20 h in the presence of different concentrations of water extract, methanol extract, or DC at 37°C. The inhibitory effects of *Neonauclea reticulata* preparations (10–500 *μ*g/mL) were dose-dependent (Figure 1) and the highest dose of *Neonauclea reticulata* extract (500 *μ*g/mL) inhibited bacterial collagenase-1 expression in 90% of cells.

### 3.3. The Antioxidant Effect of *Neonauclea reticulata* Preparations

#### 3.3.1. Scavenging of DPPH Radicals

**Figure 2:**
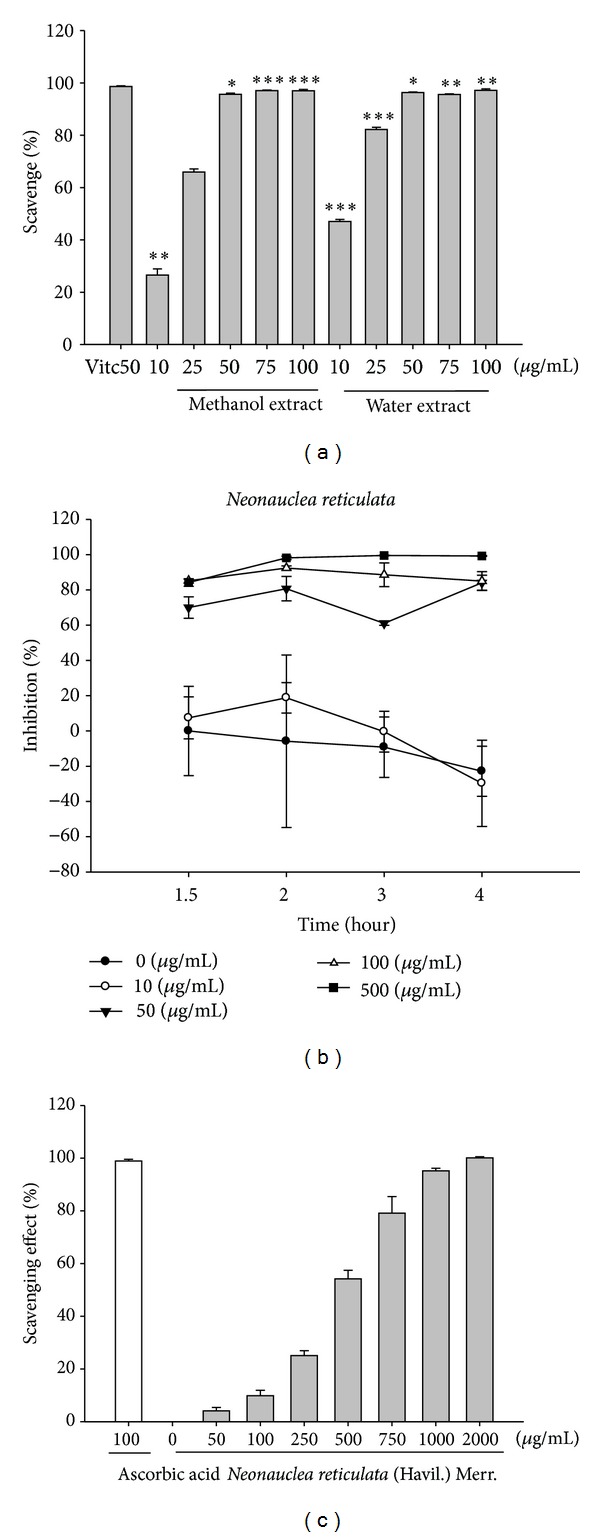
Antioxidant effect of *Neonauclea reticulata* preparation on DPPH radical scavenging and AAPH-induced hemolysis (*n* = 6). (a) Effects of methanol and water extracts of *Neonauclea reticulata* (10–100 *μ*g/mL) on DPPH radical scavenging. (b) Scavenging of hydrogen peroxide by *Neonauclea reticulata*. (c) Time course inhibition of water extract of *Neonauclea reticulata* (10–100 *μ*g/mL) on AAPH-induced hemolysis. *Neonauclea reticulata* preparations exhibited DPPH radial scavenging activity at doses higher than 50 *μ*g/mL. The IC_50_ of *Neonauclea reticulata* extract on DPPH radical scavenging was 10.2 *μ*g/mL. The *Neonauclea reticulata* extract inhibited erythrocyte hemolysis in a dose-dependent manner (10–50 *μ*g/mL). The IC_50_ of *Neonauclea reticulata* extract on AAPH-induced lysis of rat erythrocytes was 33.5 *μ*g/mL.

Figure 2(a) shows the free radical (DPPH) scavenging activity of *Neonauclea reticulata* extract (10–100 *μ*g/mL) and ascorbic acid (50 *μ*g/mL). Our results indicate that *Neonauclea reticulata* water and methanol extracts exhibited DPPH radial scavenging activity in a dose-dependent manner. The DPPH scavenging activity was significant at 10 *μ*g/mL and was similar to that of ascorbic acid at 50 *μ*g/mL (Figure 2(a)).

#### 3.3.2. Erythrocyte Hemolysis Assay

According to the results of the DPPH and MMP activity assays in a cell-free system, the activity of the water extract of *Neonauclea reticulata* was similar to those of methanol extract. Therefore, the water extract of *Neonauclea reticulata* was determined using an AAPH-induced erythrocyte hemolysis assay. The influence of the *Neonauclea reticulata* extract on *in vitro *erythrocyte hemolysis was examined by incubating rat erythrocytes in the presence of 25 mM AAPH as an initiator of oxidation. The *Neonauclea reticulata* extract exhibited a strong, dose-dependent inhibitory effect at concentrations greater than 50 *μ*g/mL (50–500 *μ*g/mL) against erythrocyte hemolysis when treated for more than 1.5 h (Figure 2(b)).

#### 3.3.3. Peroxide Scavenging Assay

Peroxide is the primary product of oxidation produced during the initial stage of oxidation. The peroxide scavenging activities of *Neonauclea reticulata* extract and ascorbic acid are shown in Figure 2(c). The mean peroxide scavenging activity of various concentrations of *Neonauclea reticulata* extract (50, 100, 250, 500, 750, 1000, and 2000 *μ*g/mL) ranged from 5.1%  ±  0.3% to 99.6%  ±  0.3% and that of ascorbic acid (1000 *μ*g/mL) was 98.5 ± 0.1%. The activity of *Neonauclea reticulata* extract was similar to that of ascorbic acid at an equal concentration (500 *μ*g/mL).

#### 3.3.4. Fluorescence Assay of Intracellular ROS

**Figure 3:**
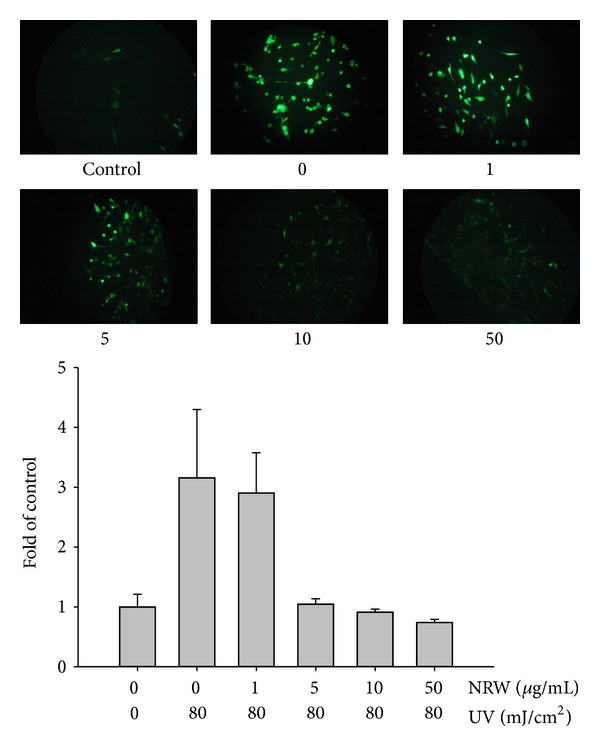
Repressive effect of *Neonauclea reticulata* extract (NRW) on intracellular oxidative stress in UV-irradiated Hs68 cells.

DCFDA staining and fluorescence microscopy were used to qualitatively characterize the degree of ROS generation. Fibroblasts were exposed to UVB (80 mJ/cm^2^) and then incubated with 10 *μ*M of DCFDA for 30 min in a 24-well plate. After removing the DCFDA-containing medium, the cells were washed with PBS and treated with *Neonauclea reticulata* extract for 24 h. As shown in Figure 3, ROS levels were markedly higher in UVB-exposed fibroblasts than in control cells; however, this increase in ROS generation was attenuated in a dose-dependent manner in UVB-exposed fibroblasts that had been pretreated with various concentrations of *Neonauclea reticulata* extract (1–50 *μ*g/mL).

### 3.4. Effect of *Neonauclea reticulata* Extract on the Cell Viability

**Figure 4:**
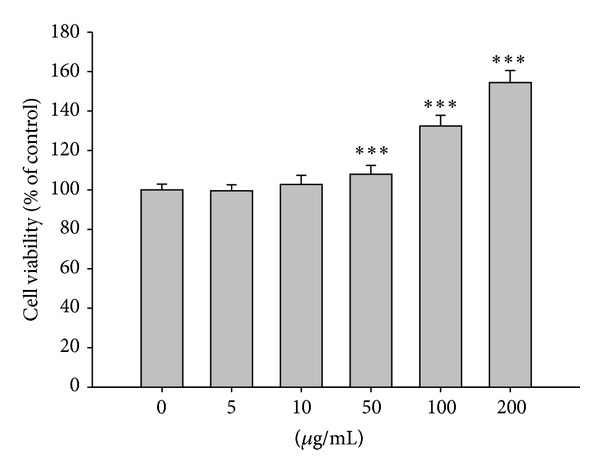
Viability (%) of human foreskin fibroblasts exposed to *Neonauclea reticulata* extract. *Neonauclea reticulata* extract (5–200 *μ*g/mL) did not exhibit cytotoxic effects on the proliferation of cells. In addition, the preparations at high concentration (>100 *μ*g/mL) stimulated cell growth (150%) (***P < 0.001).

According to the results obtained in the cell-free system mentioned above, *Neonauclea reticulata* water extract was chosen for the study in human foreskin fibroblasts (Hs68). Hs68 cells were treated with various concentrations of *Neonauclea reticulata* extract, and cell viability was measured using the MTT assay. As shown in Figure 4, the survival curve indicates that *Neonauclea reticulata* extract (5–200 *μ*g/mL) did not exhibit cytotoxic effects on the proliferation of cells. In addition, the preparations at high concentration (>100 *μ*g/mL) stimulated cell growth (140%). 

### 3.5. Effects of *Neonauclea reticulata* Extract on UVB-Induced Photoaging and the Effect of *Neonauclea reticulata* Extract on Expression of MMPs and Type I Procollagen

**Figure 5:**
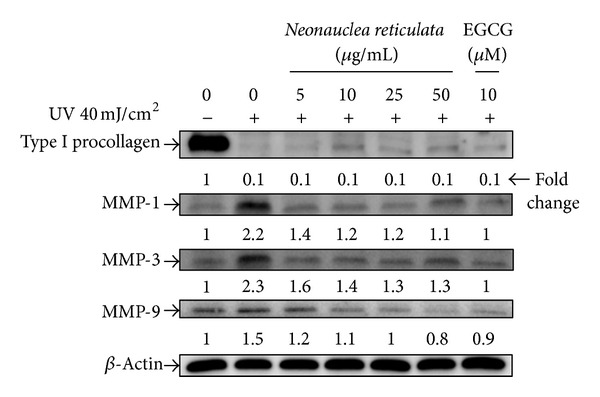
Effects of *Neonauclea reticulata* extract on UVB-induced expression of MMP-1, -3, and -9 and type I procollagen in human foreskin fibroblasts. Expression of MMPs increased and that of type I procollagen decreased after UVB irradiation. *Neonauclea reticulata* extract (5–50 *μ*g/mL) treatment attenuated this effect.

UV irradiation at a UVB dose of 40 mJ/cm^2^ resulted in overexpression of MMPs and a decrease in type I procollagen synthesis in untreated fibroblast cells (Figure 5). However, pretreatment of cells with different concentrations of *Neonauclea reticulata* before exposure to UVB (40 mJ/cm^2^) irradiation resulted in a dose-dependent reduction in expression of MMP-1, -3, -9 in cells (Figure 5). In addition, we found that UVB irradiation resulted in underexpression of type I procollagen and that pretreatment with *Neonauclea reticulata* extract did not result in a significant increase in expression of type I procollagen after exposure to UVB (Figure 5).

### 3.6. Effect of *Neonauclea reticulata* Extract on MAPK Expression

**Figure 6:**
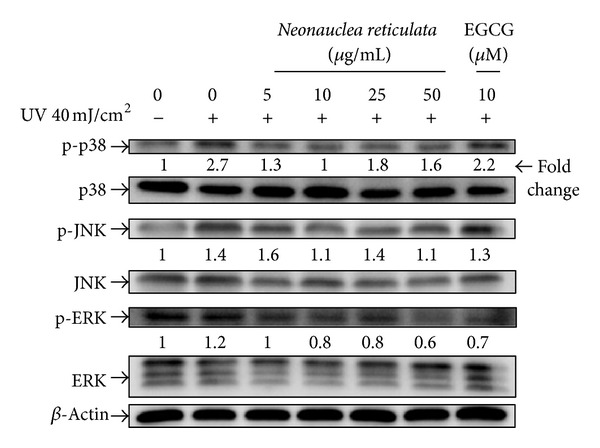
Effect of *Neonauclea reticulata* extract on UVB-induced expression of MAP kinases in human fibroblasts. *Neonauclea reticulata* extract inhibited ERK phosphorylation in a dose-dependent manner. In addition, the extract suppressed the activation of JNK at a dose of 10 *μ*g/mL. *Neonauclea reticulata* extract at low doses (5 and 10 *μ*g/mL) inhibited the phosphorylation of p38.

As shown in Figure 6, UVB (40 mJ/cm^2^) induced the phosphorylation of p38, ERK, and JNK. The inhibitory effect of *Neonauclea reticulata* extract (5–50 *μ*g/mL) on ERK phosphorylation was dose-dependent (Figure 6), and the effect was significant even at low doses (e.g., 5 *μ*g/mL). Phosphorylation of p38 and ERK was suppressed in cells that had been pretreated with *Neonauclea reticulata* extract, even at low doses.

### 3.7. Pretreatment with *Neonauclea reticulata* Extract Attenuated UVB-Induced Reduction in Total Collagen Synthesis

**Figure 7:**
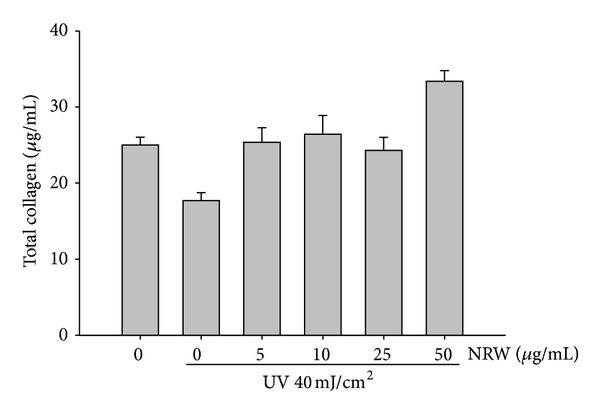
Effect of *Neonauclea reticulata* extract (NRW) on total collagen synthesis.

Fibroblasts were pretreated with *Neonauclea reticulata* extract (1–50 *μ*g/mL) for 1 h, exposed to UVB, and then treated with *Neonauclea reticulata* extract for 24 h. As shown in Figure 7, *Neonauclea reticulata* extract treatment resulted in a dose-dependent restoration of collagen.

## 4. Discussion

UVB irradiation of human skin fibroblasts induces the expression of MMPs, which in turn degrades the extracellular matrix (ECM), causing photoaging. Some investigators have, therefore, focused on the development of MMP inhibitors as a promising antiphotoaging strategy [2]. In our previous study, we demonstrated that irradiation of human skin fibroblasts with UVB caused a decrease in cell viability and type I procollagen content and an increase in ROS production and expression of MMPs [7, 8]. In this study, we found that *Neonauclea reticulata* extract not only enhanced the proliferation of fibroblasts in a concentration-dependent manner but also exhibited a protective effect against UVB-induced cytotoxicity. These results suggest that *Neonauclea reticulata* extract is a promising antiphotoaging agent.

UVB-induced inflammation and the resulting accumulation of ROS play an important role in chronologically aged and photoaged skin *in vivo* [13]. Studies have shown that intracellular generation of ROS such as superoxide anion (O_2_^−^), hydroxyl radical (OH^*∙*^), singlet oxygen (^1^O_2_), and hydrogen peroxide (H_2_O_2_) lead to a state of cellular oxidative stress and that said ROS generation is a key mediator in the photoaging process [12, 14]. The mechanisms of action of most natural product- and vitamin-based treatments that are used for skin aging involve free radical scavenging. Studies of numerous natural compounds from plant sources have shown that their photoprotective effects are mediated by their ability to quench ROS generation and prevent DNA damage due to UVB irradiation [15, 16]. In addition, other studies on natural products have reported that the high polyphenol content in these products is responsible for some of the biological activities observed in these plants. Increased cellular levels of ROS can lead to cellular damage; however, studies have shown that natural products with high polyphenol content such as *Coffea arabica*, *Terminalia catappa*, and *Emblica officinalis* protect cells from such cellular damage by scavenging ROS [7, 17–21]. In this study, we found that *Neonauclea reticulata* extract, which is abundant in polyphenols, is a natural antioxidant and that it protects against photoaging. Polyphenols are good ROS scavengers because they contain a large number of OH groups [22]. In this study, *Neonauclea reticulata* extract showed good DPPH radical and peroxide scavenging activity and protected against AAPH-induced erythrocyte hemolysis. In addition, *Neonauclea reticulata* extract exhibited scavenging activity of intracellular ROS produced by UVB irradiation, indicating that *Neonauclea reticulata* extract is a potential candidate for the prevention of aging and photoaging.

UV irradiation also enhances collagenase activity and reduces the production of collagen, resulting in wrinkle formation through degradation of the collagen in the dermal extracellular matrix [6, 23]. Collagenase inhibitors have been identified as potential therapeutic candidates for antiphotoaging and prevention of wrinkle formation [24]. MMPs cause an imbalance between collagen synthesis and degradation [5, 25]. UV irradiation induces MMP-1, MMP-3, and MMP-9 expressions [6, 26]. MMP-1 initiates the degradation of types I and III fibrillar collagens, MMP-9 further degrades collagen fragments generated by collagenases, and MMP-3 activates pro-MMP-1 [27]. Our previous studies indicated that *Coffee arabica* and *Terminalia catappa* extracts protect against photoaging induced by UVB by inhibiting the MAPK pathway and, hence, the expression of MMP-1, -3, and -9 [7, 18]. It had been reported that *Melothria heterophylla* extract and esculetin isolated from *Fraxinus chinensis* inhibited UVB-induced expression of MMP-1 mRNA and protein [28]. The results from our study indicate that *Neonauclea reticulata* extract is not only a potent MMP inhibitor but also inhibits MMP-1-induced degradation of types I and III collagen. In addition, we found that *Neonauclea reticulata* extract inhibited MMP-9 expression, thereby preventing the degradation of collagen fragments generated by MMP-1 and inhibited MMP-3 expression, which in turn resulted in a reduction in pro-MMP-1 secretion. Our findings indicate that *Neonauclea reticulata* extract promotes skin regeneration. 

MAPK activation is not only involved in photoaging but also plays a role in MMP production in fibroblasts. We found that *Neonauclea reticulata* extract inhibited JNK, ERK, and p38 activation. We speculate that the inhibition of collagen degradation by *Neonauclea reticulata* extract is related to its antioxidant activity, since the direct injury of UVB in skin is due to ROS. We also found that *Neonauclea reticulata* extract inhibits MAPK phosphorylation and causes modulation of c-Fos expression. JNK and p38 modulate c-Fos expression, and c-Fos accompanied by c-Jun synthesizes the transcription factor AP-1. The inhibition of ERK, JNK, and p38 expression by *Neonauclea reticulata* extract may also result in suppression of c-Fos and c-Jun expression, which in turn would inhibit AP-1, MMP, and type I procollagen expression. Based on our findings, we speculate that *Neonauclea reticulata* extract and its active components stimulate the proliferation of fibroblasts and TGF-*β* secretion, activate the signal transduction pathway of collagen synthesis, and suppress UVB-induced AP-1 activation.

## 5. Conclusions

**Figure 8:**
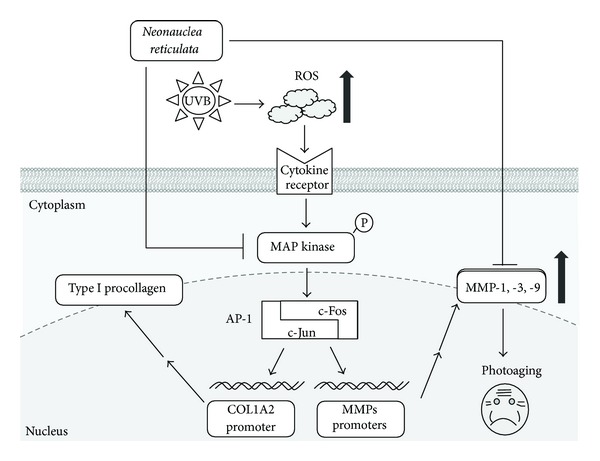
A simplified depiction of the proposed antiphotoaging mechanism of *Neonauclea reticulata* extract.

*Neonauclea reticulata* extract attenuated UVB-induced overexpression of MMP-1, -3, and -9 in fibroblasts by inhibiting UVB-induced MAPK activation (Figure 8). Our results indicate that *Neonauclea reticulata* extract is a promising antiphotoaging agent.
